# Screening of neurotransmitter receptor modulators reveals novel inhibitors of influenza virus replication

**DOI:** 10.3389/fcimb.2025.1562650

**Published:** 2025-04-29

**Authors:** Yarou Gao, Ge Liu, Yirui Ma, Yue Su, Xiaoqin Lian, Lefang Jiang, Jiaxin Ke, Xingjian Zhu, Mingxin Zhang, Yang Yu, Qun Peng, Wei Zhao, Xulin Chen

**Affiliations:** ^1^ Institute of Medical Microbiology, Department of Immunology and Microbiology, College of Life Science and Technology, Jinan University, Guangzhou, China; ^2^ Department of Biochemistry and Molecular Biology, Basic Medical College, Ningxia Medical University, Yinchuan, China; ^3^ Ningxia Clinical Research Institute, People’s Hospital of Ningxia Hui Autonomous Region, Ningxia Medical University, Yinchuan, China

**Keywords:** influenza A virus, neurotransmitter receptor, adrenergic receptors, histamine receptors, dopamine receptors, serotonin receptors, isoxsuprine

## Abstract

Influenza presents a significant public health threat, as severe cases can lead to excessive inflammation and complications such as pneumonia or acute respiratory distress syndrome. Current antiviral agents targeting viral proteins may lead to the development of resistance, highlighting the need for new agents targeting host factors. Neurotransmitter receptors are vital for cellular signaling and cell cycle modulation, making them promising antiviral therapeutic targets. Recent research has demonstrated that screening libraries of compounds aimed at these receptors can help identify inhibitors that prevent the replication of various viruses, including filoviruses and SARS-CoV-2. We screened a neurotransmitter receptor modulator library in influenza-infected U937 cells and found that many adrenergic, histamine, dopamine, and serotonin receptor agonists and antagonists exhibit antiviral activity. We identified 20 candidate compounds with IC50 values below 20 μM, suggesting a critical role for these receptors in influenza replication. Three representative compounds (isoxsuprine, ciproxifan, and rotigotine) inhibited H1N1 replication in a dose-dependent manner in multiple cell lines, and were effective against H1N1, oseltamivir-resistant H1N1, H3N2, and influenza B strains. Mechanistic studies indicated that these compounds affect virus internalization during the early infection stages. In a mouse model of lethal influenza, isoxsuprine significantly decreased lung viral titers, mitigated pulmonary inflammation, and enhanced survival rates. These findings highlight neurotransmitter receptors as potential targets for developing novel anti-influenza agents, providing a foundation for further optimization of the identified compounds as potential therapeutic agents.

## 1 Introduction

Influenza viruses pose a significant global health challenge, causing seasonal epidemics and occasionally severe pandemics that result in substantial morbidity and mortality across various populations (Taubenberger and Morens, 2008; Ge et al., 2004; Anjana et al., 2021; Horwood, 2021; Domínguez et al., 2023). Current FDA-approved antiviral medications for influenza treatment include neuraminidase inhibitors (NAIs), such as oseltamivir, zanamivir, and peramivir, as well as the polymerase acidic protein (PA) endonuclease inhibitor baloxavir marboxil (Caceres et al., 2022; Murray et al., 2023). However, these antivirals target the influenza virus directly, making it susceptible to drug resistance. The rapid evolution and antigenic drift of influenza viruses underscores the need for ongoing research to identify novel therapeutic targets and develop effective antiviral agents (Trebbien et al., 2018; Xu et al., 2024).

The influenza virus begins its life cycle by attaching via hemagglutinin (HA) proteins to sialic acid receptors on host cells (Air, 2014). The virus is then internalized mainly through clathrin-mediated endocytosis, which engulfs the virus into clathrin-coated pits that form vesicles (Edinger et al., 2014; Chen and Zhuang, 2008). These vesicles transport the virus to early endosomes, where the acidic environment in late endosomes prompts a conformational change in the HA protein, leading to fusion with the endosomal membrane (Lee, 2010). This fusion releases viral ribonucleoprotein (vRNP) complexes into the cytoplasm for replication and transcription (Staller and Barclay, 2020; Ozawa et al., 2006). Understanding this endocytosis process is crucial for developing antiviral therapies targeting viral entry.

Conventional antiviral strategies focusing on direct antiviral agents have faced challenges owing to viral adaptation, necessitating the exploration of alternative approaches that target host factors to inhibit viral replication (Tahir, 2024; Strasfeld and Chou, 2010; Shin and Seong, 2018). Recent advancements in antiviral drug discovery have highlighted the potential of targeting neurotransmitter receptors as an innovative strategy to address viral infections. For instance, Matsui et al. demonstrated that clonidine, an alpha2-adrenergic receptor (α2-AR) agonist, inhibits influenza virus replication via a cAMP-mediated pathway, improving survival in a murine model of lethal infection (Matsui et al., 2018). Similarly, studies on EBV and SARS-CoV-2 have shown promising results using neurotransmitter receptor-targeting compounds (Gorres and Hoffman, 2020; Powell et al., 2021).

Neurotransmitter receptors play crucial roles in various physiological processes, including cellular signaling pathways, such as protein kinase A (PKA), protein kinase C (PKC), neurotransmitter - gated ion channels, and cell cycle modulation (Suppiramaniam et al., 2017; Volta, 2020; Wijnen et al., 2003; Sarkar et al., 2020; ; Zdanowski et al., 2015). Their significance in essential cellular functions makes them promising targets for antiviral therapies. Recent studies have demonstrated the potential of screening compound libraries targeting these receptors to identify viral replication inhibitors, including filoviruses and SARS-CoV-2 (Cheng et al., 2015; Yang et al., 2020).

In this study, we aimed to identify inhibitors of influenza virus replication using a U937 cell-based screening approach, employing a compound library of 197 compounds that specifically target various neurotransmitter receptors. We successfully identified 20 hit inhibitors that target multiple neurotransmitter receptors. Subsequent validation and evaluation of the selected hit compounds’ efficacy, using both *in vitro* and *in vivo* models, confirmed their antiviral properties. The mechanism of action study revealed that the candidate compounds impede the internalization of the influenza virus.

Our objective was to identify novel antiviral agents by using neurotransmitter modulators and to elucidate the mechanisms through which viral replication can be disrupted. Our findings indicate that neurotransmitter receptors, including adrenergic, histamine, serotonin (5-HT), and dopamine receptors, may be involved in influenza virus replication and may serve as viable targets for the development of novel antiviral strategies. This approach offers a promising avenue to address the challenges posed by influenza viruses and other viral pathogens.

## 2 Materials and methods

### 2.1 Cell lines and virus strains

The Madin-Darby Canine Kidney (MDCK) cell (CCL-34), human lung adenocarcinoma cell line A549 (CCL-185), human monocyte cell line U973 (CRL-1593.2) were purchased from the American Type Culture Collection (ATCC, Rockville, MD, USA). MDCK cells were cultured in Dulbecco’s modified Eagle’s medium (DMEM), whereas other cells were maintained in RPMI 1640 medium. DMEM and RPMI 1640 media were supplemented with 10% fetal bovine serum (FBS, Gibco) and 1% penicillin-streptomycin. All the cells were cultured at 37°C in an incubator with 95% humidity and 5% CO_2_.

The influenza virus strains used in this study, including A/PuertoRico/8/1934 (H1N1), A/PuertoRico/8/1934 (H1N1 H274Y), A/Human/Hubei/3/2005 (H3N2), and B/Human/Hubei/1/2007 (IBV), were obtained from the virus collection at the Wuhan Institute of Virology, Chinese Academy of Sciences, China.

### 2.2 Chemicals

A customized neurotransmitter receptor-targeting small-molecule compound library was purchased from Selleck Chemicals (Shanghai, China) for anti-influenza drug screening. The library comprises 197 compounds that target a range of receptors, including adrenergic, 5-hydroxytryptamine(5-HT), acetylcholine, histamine, dopamine, γ-aminobutyric acid type A(GABA), glutamate, and opioid receptors.

We selected three hit compounds, isoxsuprine, ciproxifan, and rotigotine, for further *in vitro* and *in vivo* antiviral efficacy studies and purchased them from MedChemExpress Co., Ltd. (Shanghai, China). Ribavirin was purchased from Sigma-Aldrich (St. Louis, MO). Oseltamivir (GS 4071) was purchased from Toronto Research Chemicals (Toronto, Canada). All the compounds were dissolved in DMSO (Sigma-Aldrich) to a final concentration of 10 mM. DAPI was purchased from Solarbio (Beijing, China).

### 2.3 Antibodies

Mouse monoclonal antibodies against influenza A virus nucleoprotein (NP) were purchased from Sino Biological (Beijing, China). Goat anti-mouse IgG (H+L) conjugated with Alexa Fluor^®^488 (green) was purchased from Cell Signaling Technology Inc. (MA, USA). Mouse monoclonal antibodies against GAPDH, HRP-labeled goat anti-mouse IgG (H+L), and HRP-labeled goat anti-rabbit IgG (H+L) were purchased from Beyotime Biotechnology (Shanghai, China).

### 2.4 Neuraminidase activity assay

2’-(4-Methylumbelliferyl)-a-D-N-acetylneuraminic acid (MUNANA, Sigma, M8639) is a fluorescent substrate for influenza virus neuraminidase (NA) used to detect the amount of infectious virions in cell culture supernatants (An et al., 2014). The culture supernatant containing the virus was transferred to a black opaque 96 or 384 well plate (PerkinElmer, 6005270 or 6007270, respectively), mixed with 20 μmol/L MUNANA in MES solution (33 mmol/L 2-[N-morpholino] ethanesulfonic acid and 4 mmol/L CaCl_2_; pH 6.5) and incubated at 37°C for 1 h. The reaction was stopped by adding stop solution (0.14 M NaOH in 83% ethanol). Fluorescence intensity was measured at excitation and emission wavelengths of 365 and 445 nm, respectively, using a multilabel plate reader (Envision2103, PerkinElmer, USA).

### 2.5 Cell viability assay

The CellTiter 96 Aqueous One Solution Cell Proliferation Kit (Promega, G3580) was used to assess cell viability. Cells were treated with or without the compound for 48 h. Following removal of the supernatant (170 μL in 96-well plates and 70 μL in 384-well plates) from the U937 cells, a medium (RPMI 1640 with 10% FBS) containing 20% detection reagent was added and incubated at 37°C for 1 h. The test plate was centrifuged at 5000 × g for 3 min with a swing-out rotor, and the optical density (OD) at 490 nm (OD490) and reference wavelength at 630 nm (OD630) were measured using an EnVision Multilabel Plate Reader (PerkinElmer, MA, USA). The final absorbance was calculated by subtracting OD630 from OD490 to eliminate the effects of cell debris, fingerprints, and other non-specific absorption.

### 2.6 Screening of inhibitor library against influenza virus infection

In the primary screening, 197 compounds (10 mM) were added to 384-well source plates (LabCyte, LP-0200). Each compound, the positive control drug, or DMSO (160 nL) was transferred to four 384-well plates (PerkinElmer, 6007460) using an acoustic droplet ejection system (Echo 550, Labcyte, CA, USA), followed by the addition of complete medium (40 µL; RPMI 1640 with 10% FBS and 1% penicillin-streptomycin) to dilute the drug to 20 μM.

U937 cells were resuspended at 1×10^6^cells/mL in a complete medium and infected with 0.05 multiplicities of infection (MOI) A/PuertoRico/8/1934 (H1N1) virus. Subsequently, 40 μL of the cell-virus mixture was added to each compound-containing well at 40,000 cells/well, and 40 μL of uninfected cells was added to the negative control wells. After a 48-hour incubation at 37°C and 5% CO_2_/95% relative humidity, cell culture plates were centrifuged at 5000 × g for 3 min, and 70 μL of supernatant per well was used for the NA activity assay. The remaining cells were used to assess the cell viability. Hits are compounds that inhibit viral production and cell viability by more than 50% and 85%, respectively. All hit compounds were subjected to validation screening.

In the validation screen, serially diluted hit compounds (0.04–80 μM) were added to 384-well plates. U937 cells and influenza virus were added and incubated for 48 h to confirm the inhibitory effect and study drug response kinetics. Cytotoxicity was determined under the same conditions without viral infection. Finally, the hit compounds’ inhibitory concentration 50% (IC50), cytotoxic concentration 50% (CC50), and selective index (SI) were determined.

### 2.7 Measurement of virus titer

Viral titers were determined using the TCID50 assay. Briefly, MDCK cells were seeded into 96-well cell culture plates at a density of 20, 000 cells/well. 24 h later, tenfold serial dilutions of the virus-containing supernatants were inoculated onto an MDCK monolayer at 37°C for 72 h, and cytopathic effects (CPE) were examined. Virus titers were calculated using the method developed by Reed and Muench (Reed and Muench 1938).

### 2.8 Virucidal assay

Virucidal activities of the compounds were evaluated using a virucidal assay. Briefly, influenza virus A/PuertoRico/8/1934 (2×10^7^ pfu/ml) was incubated with each compound at 5 times its IC50 for 3 h at 37°C, diluted 100-fold, and used to infect A549 cells (MOI=1). For the control groups, the same amounts of virus and compounds were incubated separately at 37°C for 3 h, diluted 50-fold, and mixed immediately to infect the cells. At 12 h post-infection, supernatants were collected for NA activity assays to determine the percentage of inhibition. Tannic acid served as a positive control and DMSO was used as a solvent control.

### 2.9 Time of addition assay

The antiviral activities of the compounds were evaluated at different stages of influenza virus infection using time-of-addition assay. These stages included cell pretreatment, virus adsorption, and post-infection. Specifically, confluent A549 cells were treated with each candidate compound at five times the IC50. For pretreatment, cells were incubated with the compounds for 2 h at 37°C before infection. In the adsorption group, compounds were present only during the 1 h adsorption period at 4°C. In the post-infection treatment, the compounds were added immediately after the adsorption period at the indicated time points. Supernatants were collected 12 h post-infection and neuraminidase (NA) activity was measured.

### 2.10 Indirect immunofluorescence assay (IFA)

A549 cells were washed twice with PBS and fixed with 4% paraformaldehyde (PFA) for 20 min at room temperature (RT). After three washes with PBS, cells were permeabilized with 0.25% Triton X-100 in PBS for 15 min at room temperature. Permeabilized samples were blocked with 5% BSA in PBS at 37°C for 1 h and then incubated with mouse anti-NP antibody at 4°C overnight. After three washes with PBS, cells were incubated with Alexa Fluor 488 goat anti-mouse IgG for 1 h. The nuclei were stained with DAPI for 5 min after three additional washes. After three final washes with PBS, immunofluorescence was analyzed using an Operetta CLS high-content analysis system (PerkinElmer).

### 2.11 Viral binding and internalization assays

For the viral binding assay, A549 cells were pre-chilled at 4°C for 1 h, and 1 mL of virus solution containing different concentrations of the compounds was added. After adsorption at 4°C for 1 h, the cells were washed 3 times with cold PBS (pH 7.2) or cold acidic PBS (pH 1.3), the cells were lysed with 1 x SDS-PAGE loading buffer (Beyotime Biotechnology, Shanghai, China) and subjected to Western Blotting for NP detection.

For the viral internalization assay, A549 cells were pre-chilled at 4°C for 1 h, and 1 mL of virus solution containing different concentrations of the compounds was added. After adsorption at 4°C for 1 h, the cells were transferred to 37°C for 30 min to allow internalization. The cells were washed 3 times with ice-cold acidic PBS (pH 1.3) to remove the attached but not-yet-internalized virions and then subjected to Western Blotting for NP detection.

### 2.12 Western Blotting assay

Cells were washed twice with ice-cold PBS and lysed using RIPA buffer (Beyotime Biotechnology, Shanghai, China). The cell lysate was centrifuged at 4°C for 10 minutes at 12000 x g, and the supernatants were collected. The supernatants were boiled for 10 min, and 10 µg protein of the cell lysate was loaded onto an SDS gel. The polyvinylidene fluoride (PVDF) membranes were blocked with 5% skim milk in TBST and incubated at room temperature for 1 h. The membranes were then incubated overnight at 4°C with primary antibodies against influenza NP (1:1000) and GAPDH (1:1000). The membranes were then treated with an HRP-conjugated secondary antibody (1:2000), and the target proteins were visualized using Clarity Western ECL Substrate (Bio-Rad, Singapore). The signals were captured using a ChemiDoc imaging system (Bio-Rad).

### 2.13 Animal experiment

The compounds were resuspended in 0.9% sodium chloride containing 5% DMSO. Female BALB/c mice (6 weeks old, 10 per group) received either placebo or isoxsuprine (40, 80, or 160 mg/kg/day) 30 min before being infected with the mouse-adapted influenza virus A/Puerto Rico/8/1934 (H1N1) at a dose 10 times the LD50 in PBS. The mice were administered 0.9% sodium chloride with 5% DMSO or isoxsuprine intraperitoneally twice daily for three consecutive days. After infection, all the mice were monitored daily for 21 days for survival, weight loss, and clinical signs of illness. The lung tissue was collected on days 5 and 7 post-infection to determine the virus titers using the TCID50 assay. Hematoxylin and eosin (H&E) staining was performed as previously described. All mouse lung tissues were fixed in 4% paraformaldehyde overnight at day 5 post-infection, embedded in paraffin, and cut into 4 µm sections for H&E staining. H&E-stained slides were examined for tissue damage, necrosis, and infiltration of inflammatory cells.

### 2.14 Preparation of lung tissue homogenates

Mice were humanely euthanized, and the lung tissue was aseptically harvested. The lung tissue was placed in a centrifuge tube along with an appropriate volume of sterile phosphate-buffered saline (PBS, pH 7.0), typically 1 to 2 mL per 100 mg of tissue. Using a tissue grinder, the sample was homogenized thoroughly on ice. Subsequently, the homogenate was subjected to centrifugation at 8000 g for 15 minutes at a temperature of 4°C. The resulting supernatant was carefully transferred to a new tube and stored at -80°C for subsequent measurement of virus titer.

### 2.15 Statistical analyses

The concentrations necessary to inhibit viral replication by 50% (IC50) and reduce cell viability by 50% (CC50) and the selective indices (SIs, defined as CC50/IC50) of the compounds were determined using Prism v8.0 software (GraphPad Software, San Diego, CA, USA). Data are presented as the mean ± standard error of the mean (SEM) for each measurement. Differences between the averages of the control and test samples were statistically analyzed using the Student’s t-test, with statistical significance set at P < 0.05, statistically significant (*). p < 0.01, highly statistically significant (**). p < 0.001, very highly statistically significant (***). A two-way repeated-measures ANOVA with *post-hoc* Bonferroni’s t-test was conducted for body weight studies. The survival rates of mice were compared using the log-rank (Mantel-Cox) test.

## 3 Results

### 3.1 The screening identifies 20 compounds that inhibit the influenza virus replication in U937 cells

To investigate the effect of inhibitors targeting neurotransmitter receptors on influenza virus replication, we conducted a primary screening of 197 compounds, including antagonists, agonists, and inhibitors of neurotransmitter receptors, using a cell-based model specified in U937. Our findings identified 28 hit compounds that achieved over 50% inhibition of virion production, as evaluated by measuring neuraminidase activity in cell culture supernatants. Importantly, most of these compounds maintained a cell viability above 85% at a concentration of 20 μM (**Supplementary Table 1**).

**Figure 1:**
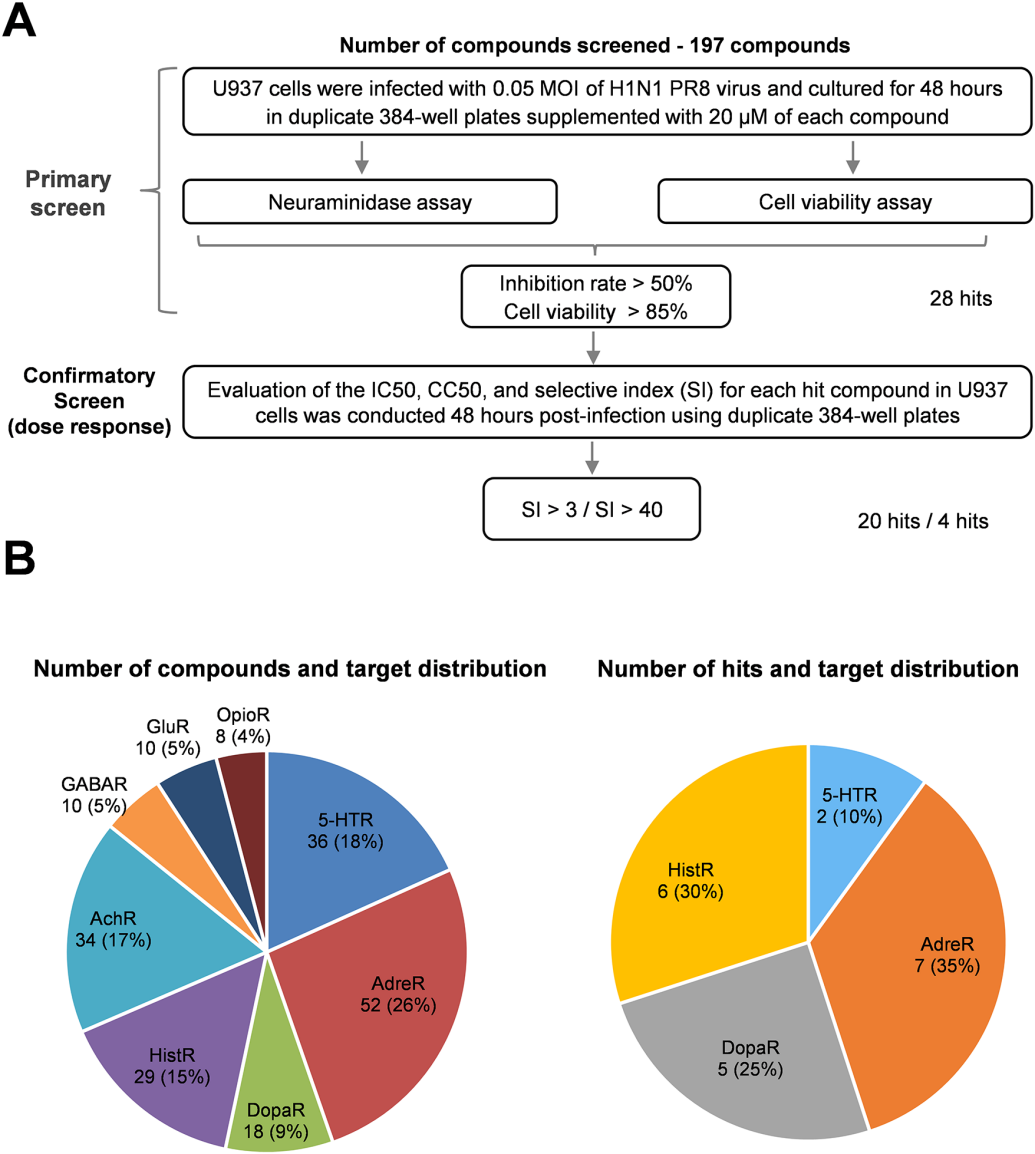
Screening of a neurotransmitter receptor-related compounds library for anti-influenza drugs. **(A)** Flow chart of HTS of a neurotransmitter receptor-related compound library of anti-influenza drugs. The flow chart includes the steps and conditions for the entire screening process and lists the selection criteria for the hit compounds and the number of hits obtained after each screening. **(B)** Pie charts represent the library of compound receptor types (left) and confirmed hits (right). Each percentage in the pie chart indicates the ratio of the number of specific receptor-related compounds to the total number of compounds. AdreR, Adrenergic Receptors; 5-HTR, 5-hydroxytryptamine receptors; AChR, Acetylcholine Receptor; HistR, Histamine Receptors; DopaR, Dopamine Receptors; GABAR, γ-aminobutyric acid type A receptors; GluR, Glutamate Receptors; OpioR, Opioid Receptors.

Following primary screening, we performed a validation assay to evaluate the inhibition of influenza virus infection and assess cytotoxicity. We applied serial concentrations of each identified compound to infected cells at a maximum drug concentration of 80 μM because of the limited availability of each compound in our library. Consequently, the CC50 values for many hit compounds could not be finalized and were labeled >80 μM. After confirmatory screening, we identified 20 compounds with a selective index of more than 3 and four compounds with a selective index exceeding 40 (**Figure 1A**). The antiviral properties and drug targets of the candidate compounds were listed in **Table 1**.

**Table 1:** Hits confirmed to have antiviral activity against influenza virus infection.

Chemical name	Receptor Type	Target & Possible mechanisms^a^	CC_50_	IC_50_	SI
Medetomidine HCl	Adrenergic receptor	α2-receptor agonist	>80	12.5	>6.4
Phentolamine Mesylate		α-receptor antagonist	>80	1.0	>80.0
Nebivolol		β1-receptor antagonist	33.3	9.8	3.4
Salmeterol Xinafoate		β2-receptor agonist	40.2	4.4	9.1
Isoxsuprine		β2-receptor agonist	>80	1.5	>47.1
ICI-118551		β2-receptor antagonist	>80	5.8	>13.8
Propranolol HCl		β-receptor antagonist	>80	13.9	>5.8
Clemastine Fumarate	Histamine receptor	H1-receptor antagonist	65.3	4.9	13.3
Meclizine 2HCl		H1-receptor antagonist	79.5	5.3	15
Azelastine HCl		H1-receptor antagonist	55.7	8.4	6.6
Promethazine HCl		H1-receptor antagonist	>80	9.3	>8.6
Lafutidine		H2-receptor antagonist	>80	14.8	>5.4
Ciproxifan		H3-receptor antagonist	>80	1.4	>57.1
SB269970 HCl	5HT	5-HT7-receptor antagonist	>80	2.8	28.6
Dapoxetine HCl		SSRI^b^	76.1	6.65	11.4
SKF38393 HCl	Dopamine receptor	D1/D5-receptor agonist	>80	8.5	>9.4
Prochlorperazine dimaleate		D2-receptor antagonist	38.4	8.3	4.63
Pergolide mesylate		D-receptor agonist	>80	7.1	>11.3
Rotigotine		D-receptor agonist	>80	1.26	>63.5
Benztropine mesylate		DAT^c^ Inhibitor	>80	19.2	>4.2

In this table, the 50% cytotoxic concentration (CC50, μM) of the hits was determined via cell viability assays. The 50% inhibition concentration (IC50, μM) was measured through neuraminidase activity assays. The Selective Index (SI) represents the ratio of CC50 to IC50. Notes,

^a^ The target and function of the compound as indicated on its accompanying label or literature.

^b^ SSRI, Selective serotonin reuptake inhibitor.

^c^ DAT, Dopamine transporter.

Notably, the identified compounds interacted with adrenergic receptors (seven hits), histamine receptors (six hits), 5-HT receptors (two hits), and dopamine receptors (five hits), as shown in **Figure 1B** and summarized in **Table 1**. However, no hits were found among compounds targeting acetylcholine, γ-aminobutyric acid type A, glutamate, and opioid receptors despite representing 62 compounds constituting 31% of the library (**Figure 1B**).

The results indicate that The IC50 values for most of the identified compounds range between 1 and 20 μM, suggesting that the inhibitors targeting neurotransmitter receptors exhibit significant antiviral activity against influenza virus infection. Our findings reveal that a few candidate compounds are toxic to rapidly proliferating monocytic U937 cells, with a CC50 value of approximately 50 μM. However, most hit compounds exhibit low cytotoxicity, with CC50 values exceeding 80 μM (**Table 1**).

### 3.2 The selected hit compounds exhibit antiviral effects in multiple cell lines

**Figure 2:**
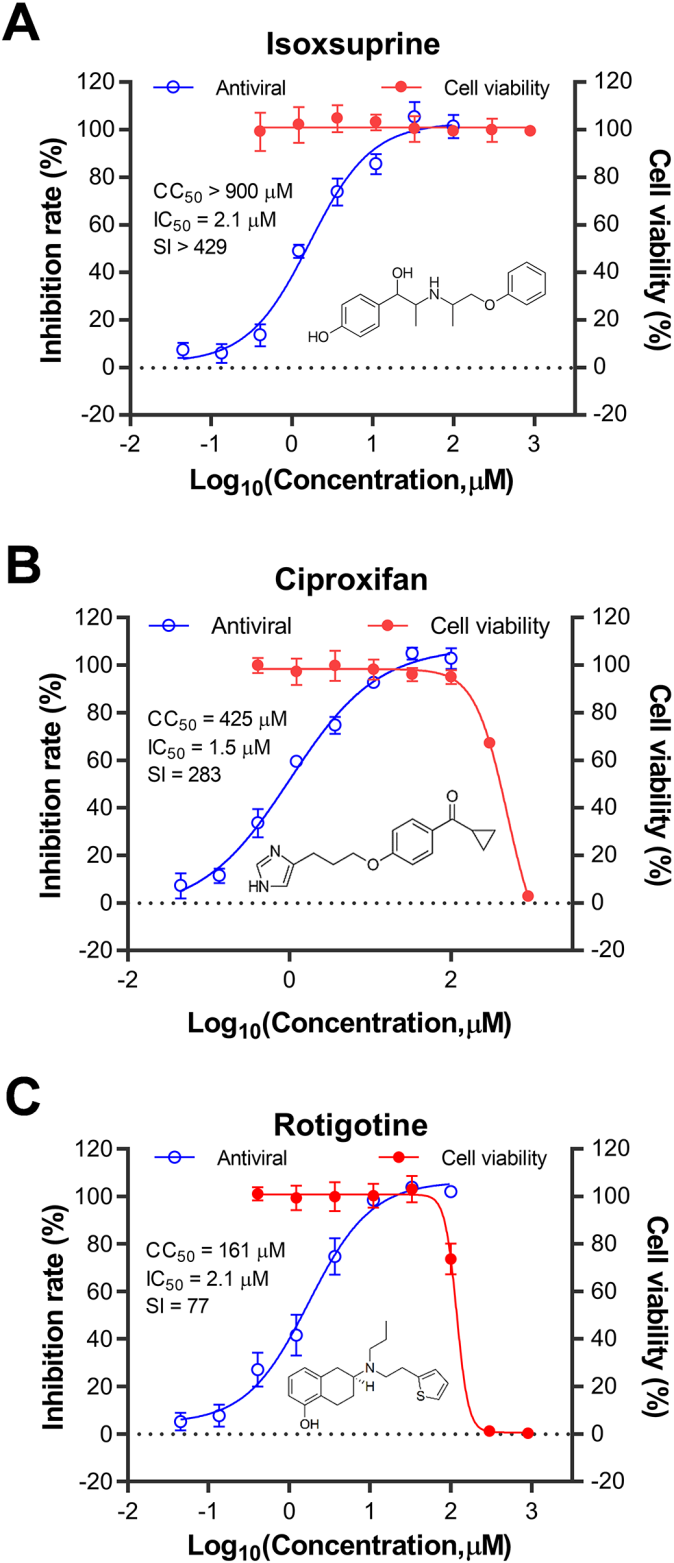
Kinetic curves illustrating the toxicity and antiviral activity of the identified compounds in U937 cells. The compounds tested for drug toxicity and antiviral activity were isoxsuprine **(A)**, ciproxifan **(B)**, and rotigotine **(C)**. The U937 cells were seeded in 96-well plates at a density of 1 × 10^6 cells/mL in complete medium and then infected with A/Puerto Rico/8/1934 (H1N1) virus at a multiplicity of infection (MOI) of 0.05. Structures of the three compounds are shown in each panel. The antiviral activity of the candidate compounds was measured by assessing the inhibitory rate of neuraminidase (NA) activity in the supernatant collected from each treatment group. At the same time, the cytotoxicity of these compounds was evaluated in uninfected U937 cells using a cell viability assay. The data presented represent the results from three independent experiments, expressed as mean ± Standard Error of the Mean (SEM). The CC50 and IC50 values were calculated using GraphPad Prism software (version 8.0).

Three compounds were selected to assess their antiviral activities against the influenza virus in various cell types capable of supporting viral replication. These compounds were sourced from a vendor different from the drug library to avoid any potential errors from using degraded or incorrect substances. As shown in **Figure 2**, the compounds evaluated were isoxsuprine (a β2-adrenergic receptor agonist), ciproxifan (an H3-histamine receptor antagonist), and rotigotine (a D-dopamine receptor agonist). Their antiviral activity against the influenza A virus (IAV) PR8 strain in human U937 cells was determined by measuring the neuraminidase (NA) activity in the supernatant. The corresponding IC50 values were 2.1 μM for isoxsuprine, 1.5 μM for ciproxifan, and 2.1 μM for rotigotine. Concurrently, the cytotoxicity of these compounds in uninfected U937 cells was evaluated by cell viability assay. The CC50 values for the selected compounds were >900 μM, 425 μM, and 161 μM for isoxsuprine, ciproxifan, and rotigotine, respectively. The calculated selective indices for these three compounds were >429, 283, and 77, respectively, indicating that they exhibit highly potent antiviral activity against the PR8 influenza virus replication.

To evaluate whether the antiviral effects of the selected compounds were cell type-dependent, we investigated their antiviral activities against the same strain of influenza virus in human A549 and Madin-Darby canine kidney (MDCK) cells. The results summarized in **Table 2** indicate that the three selected compounds exhibit comparable cytotoxicity and antiviral activity in A549 cells relative to those observed in U937 cells. Notably, the three compounds demonstrate significantly lower antiviral activity in MDCK cells, as evidenced by their higher IC50 values compared to the other two human cell lines, despite exhibiting similar cytotoxicity. These three compounds exhibit considerably lower selectivity indices (SIs) in canine cells. Nevertheless, the three identified hit compounds demonstrate robust antiviral activity against influenza virus replication regardless of the cell type examined.

**Table 2:** Antiviral activities of the selected hit compounds against influenza virus A/PuertoRico/8/1934 (H1N1) on different cells. In this table, the 50% inhibition concentration (IC50) of the candidate hits against the virus was determined using a neuraminidase activity assay. The 50% cytotoxic concentration (CC50) was measured via cell viability assays. The Selective Index (SI) is calculated as the ratio of CC50 to IC50, providing an indication of the compounds' selectivity between antiviral and cytotoxic effects across different cell types.

Compound Name	U937			A549			MDCK		
	IC50 (µM)	CC50 (µM)	SI	IC50 (µM)	CC50 (µM)	SI	IC50 (µM)	CC50 (µM)	SI
Isoxsuprine	2.1	>900	>429	4.9	>900	>184	36.3	>400	>11
Ciproxifan	1.5	425	283	6.3	556	88.3	59.4	>400	>7
Rotigotine	2.1	161	77	14.7	195	13.3	8.1	93	11

### 3.3 The selected compounds have broad-spectrum antiviral effects against influenza virus infection

**Figure 3:**
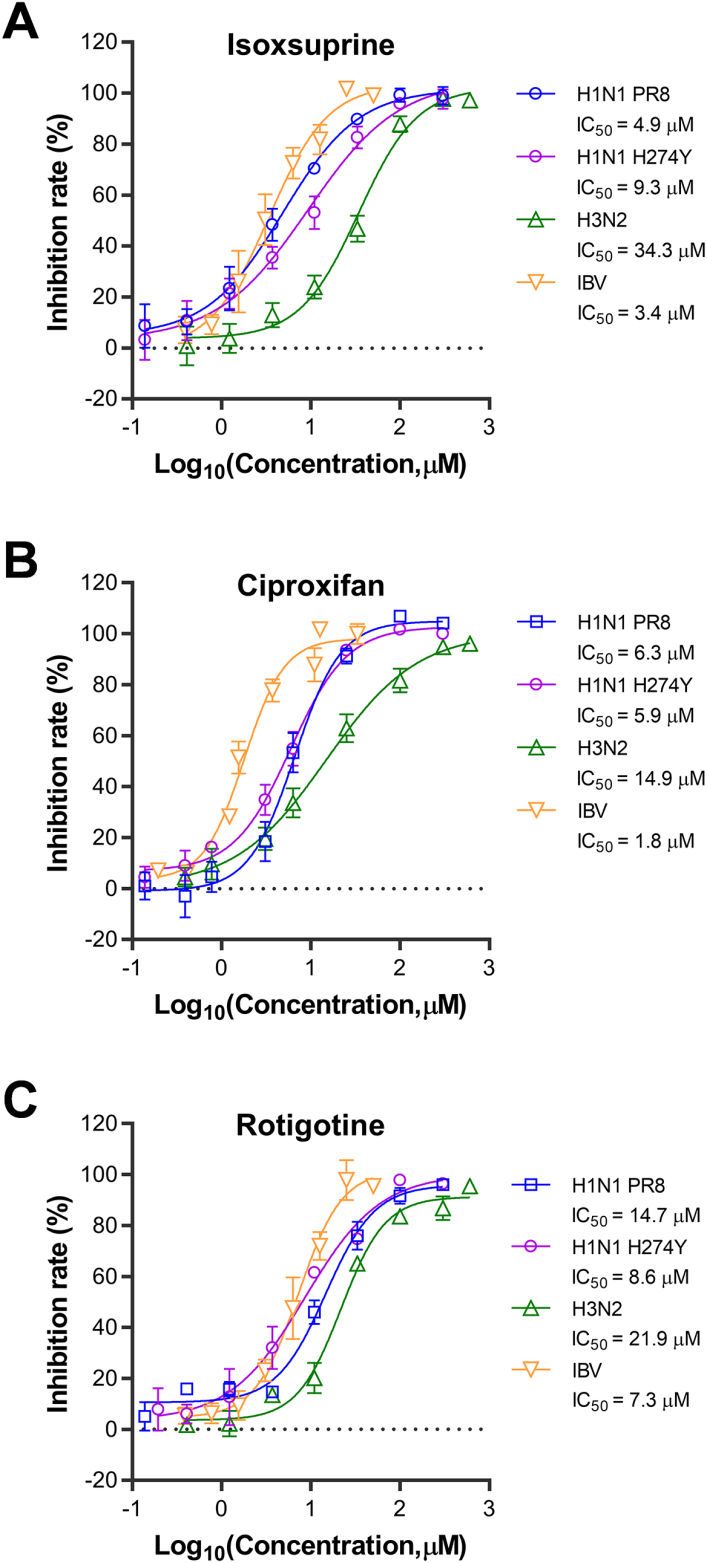
Broad-spectrum antiviral effects of the three selected compounds against different strains of the influenza virus. compounds tested for broad-spectrum antiviral effects were isoxsuprine **(A)**, ciproxifan **(B)**, and rotigotine **(C)**. The cells used for H1N1 PR8, H1N1 H274Y, and H3N2 infections were A549 cells, while MDCK cells were used for IBV infection. The cells were trypsinized and resuspended for 24 h before infection. They were then seeded in 96-well plates at 20,000 cells per well for A549 cells and 15,000 cells per well for MDCK cells. Once the cells reached approximately 80% confluence, they were infected with 0.1 MOI of the respective viruses in the presence of varying concentrations of the compounds. After 48 h of incubation at 37°C, supernatants were collected, and the viral inhibition rate was determined using a neuraminidase (NA) activity assay. The data presented represent results from three independent experiments (mean ± SEM). The IC50 values were determined using GraphPad Prism software (version 8.0).

The dynamic characteristics of influenza viruses, characterized by their rapid evolution and antigenic drift, result in variable responses to antiviral agents across subtypes and drug-resistant strains. To evaluate the broad-spectrum antiviral efficacy of the three selected compounds, we assessed their antiviral activities against three additional influenza virus strains: H1N1, oseltamivir-resistant H1N1 (H274Y), H3N2, and IBV. As shown in **Figure 3**, the results indicate that the antiviral effects of the three compounds against oseltamivir-resistant H1N1 (H274Y) in A549 cells are similar to those observed against wild-type H1N1. Ciproxifan’s IC50s are comparable, but isoxsuprine and rotigotine show a two-fold difference in IC50s. However, the antiviral activities of these compounds are less pronounced against the H3N2 IAV strain in A549 cells than their effects on both wild-type and oseltamivir-resistant H1N1 strains, with 7.0, 2.4, and 1.5-fold higher in IC50s for isoxsuprine, ciproxifan, and rotigotine, respectively. The antiviral effects on the IBV strain were assessed using MDCK cells, in which IBV displayed more efficient replication than in A549 cells. Notably, despite differences in subtypes, similar antiviral effects were observed on the IBV strain compared to those on IAV H1N1, with a ratio of 0.7, 0.3, and 0.5 in IC50s for isoxsuprine, ciproxifan, and rotigotine, respectively. In conclusion, the three selected compounds exhibit broad-spectrum antiviral activity against various subtypes and drug-resistant influenza virus strains.

### 3.4 The selected compounds inhibit the early stage of influenza virus replication

**Figure 4:**
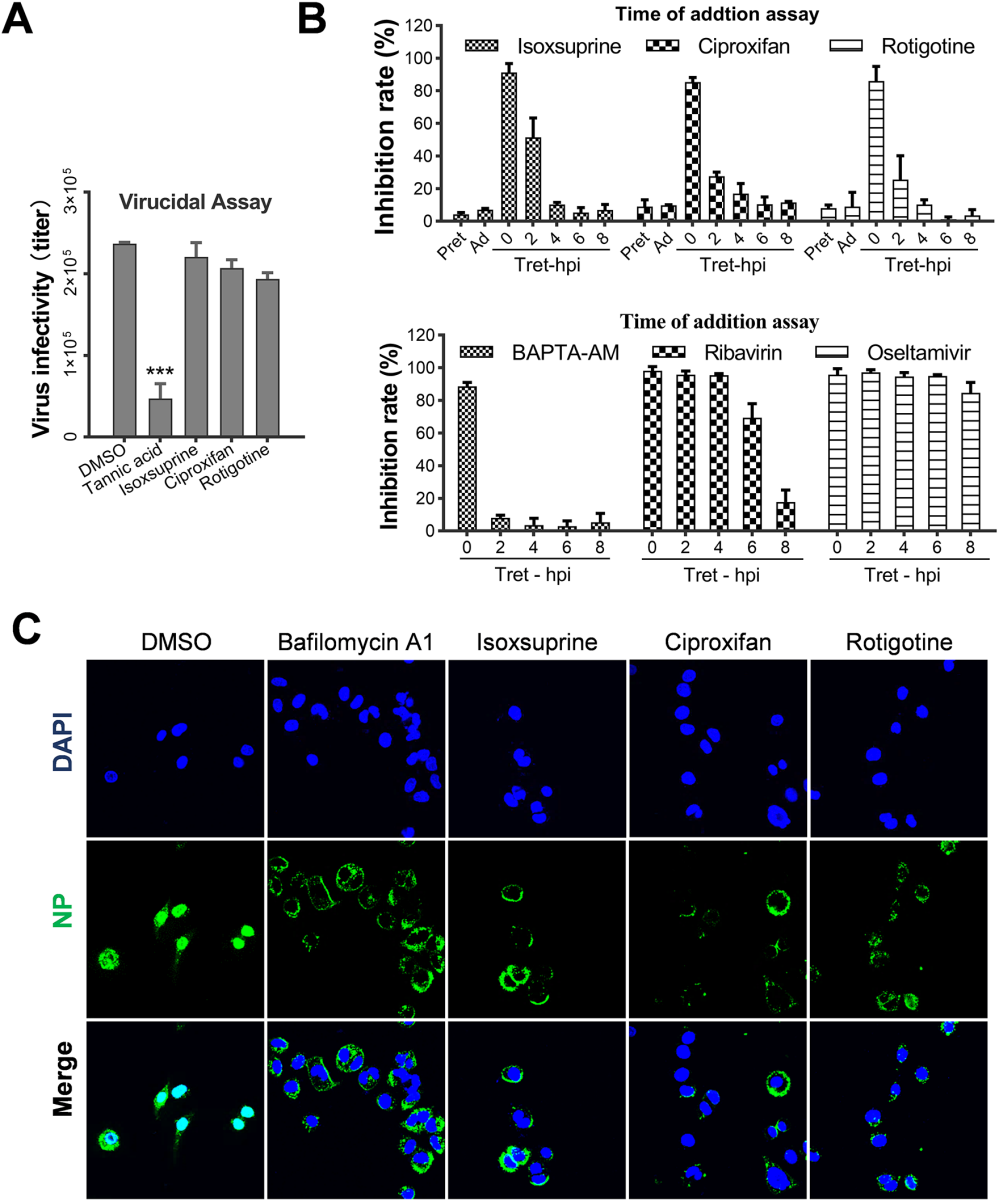
Selected hit compounds impair the early stages of the influenza virus life cycle. **(A)** Virus inactivation assay. Influenza virus PR8 (2×10^7^ pfu/ml) was incubated with 5 IC50 of each candidate at 37°C for 3 h and then diluted 100-fold and used to infect A549 cells (MOI=1). In the control groups, equal amounts of virus and drugs were incubated separately at 37°C for 3 h, diluted 50-fold, and mixed to infect the cells. The final concentrations of the candidates were identical between the two groups and ineffective against IAV replication. At 12 h p.i., the inhibitory rate based on neuraminidase (NA) activity in the supernatants was measured. ***, p < 0.001. **(B)** Time of addition assay. Confluent A549 cells were treated with 5 IC50 of each candidate at 37°C for 2 h before infection (pretreatment group, pre), during the adsorption period at 4°C for 1 h (adsorption group, ad), or at the indicated times immediately after absorption treatment (post-infection groups). At 12 h p.i., the inhibitory rate based on neuraminidase (NA) activity in the supernatants was measured. Values represent the mean ± SEM. of duplicate samples from three independent experiments. **(C)** A549 Cells were infected with PR8 at an MOI of 10 and treated with the I5C50 values of each candidate at 37°C. At 4 h p.i., confocal microscopy was used to detect viral NP and nuclear colocalization. Nuclei were labeled with DAPI, and Bafilomycin A1 was used as a positive control.

Prior to the study of the antiviral mechanism, a virucidal assay was performed to assess the candidate compounds’ ability to inhibit influenza virus infectivity. As shown in **Figure 4A**, direct treatment with the three candidate compounds did not significantly reduce influenza virus infectivity. In contrast, treatment with the reference compound tannic acid resulted in a substantial decrease in viral infectivity of over one log unit.

Subsequently, to elucidate the mechanism by which the selected candidate compounds inhibit the influenza virus replication cycle, a time-of-addition assay was conducted to identify the specific stage of viral replication. As shown in **Figure 4B**, neither pretreatment of cells (pre) nor treatment during the virus adsorption inhibited viral replication. However, early administration of each candidate compound post-virus infection substantially inhibited viral replication. Conversely, when administered two hours post-infection, the compounds did not exhibit any inhibitory effect. Concurrently, reference drugs BAPTA-AM, ribavirin, and oseltamivir, which target internalization, RNA replication, and virion release respectively, were evaluated for their antiviral effectiveness using the time-of-addition assay. As anticipated, these agents inhibited the early, middle, and late stages of viral replication. Consistent with the action of BAPTA-AM, which obstructs the internalization of the influenza virus, isoxsuprine, ciproxifan, and rotigotine exhibited inhibition when administered during the initial stages of viral replication. This observation indicates that these compounds exert their effects at this early stage of the replication cycle.

Furthermore, an immunofluorescence assay (IFA) was performed to determine whether the import of viral ribonucleoprotein (vRNP) was disrupted by drug treatment. As shown in **Figure 4C**, in untreated cells, vRNPs were successfully imported into the nuclei, resulting in the expression and nuclear import of new viral nucleoproteins (NP), which produced a bright fluorescence signal. Conversely, in drug-treated cells, vRNP import was completely inhibited, as evidenced by the absence of NP in the nuclei of infected cells. Instead, NP associated with virions were localized around the cell membranes, suggesting that the internalization process or fusion of the viral and endosomal membranes is impeded, leading to failure of vRNP release and subsequent nuclear import.

In conclusion, the three candidate compounds impede the early stages of influenza viral replication, rather than exerting a virucidal effect. This implies that one or more steps, including viral attachment, internalization, acidification, and membrane fusion, are blocked.

### 3.5 The selected compounds inhibit the replication of influenza viruses by blocking virus internalization

**Figure 5:**
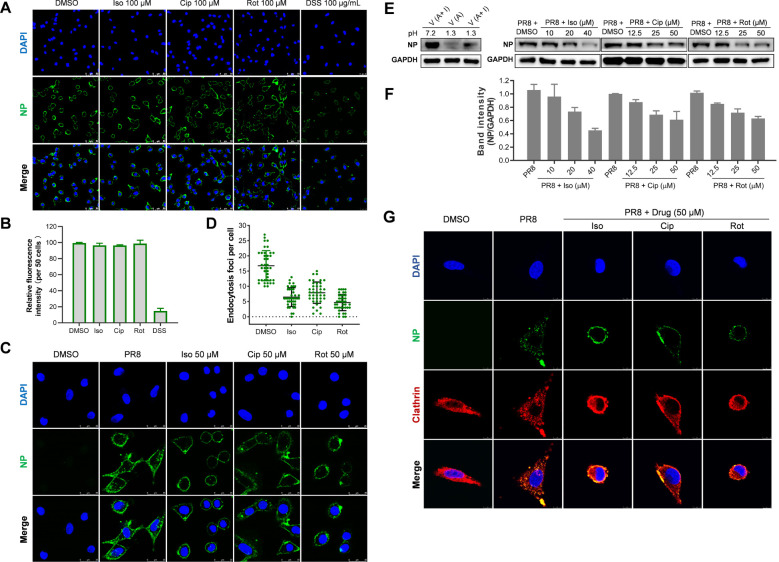
Selected hit compounds inhibit internalization of the influenza virus. **(A)** Virus Attachment Assay: Cells were precooled at 4°C for 30 minutes and then infected with PR8 (MOI = 10) in the absence or presence of each candidate compound at the indicated concentrations for 1 hour at 4°C. The cells were then fixed with 4% paraformaldehyde (PFA). The viral NP was fluorescently stained green, and the nuclei were stained blue with DAPI. Images were captured using a Leica SP8 confocal microscope. Scale bar: 50 µm. **(B)** The total fluorescence intensities per field in A were quantified using ImageJ software; the result is one representative IFA data set. **(C)** Indirect Immunofluorescence Assay (IFA): Following viral attachment (MOI = 25) at 4°C for 1 hour, the cells were transferred to 37°C for 1 hour to allow for internalization in the absence or presence of each candidate compound at the specified concentrations. The level of virus internalization was measured using IFA. Scale bar: 25 µm. **(D)** The quantitative endocytosis in **(C)** was determined by counting the number of NP fluorescent foci in the cytoplasm per cell. Values are expressed as the means ± SEM of two independent experiments. **(E)** Internalization of influenza virus determined by acid treatment and Western botting Analysis: After viral attachment (MOI = 5) at 4°C for 1 hour, cells were washed twice with cold PBS (pH 7.2). One group underwent three washes with cold PBS-HCl (pH 1.3) to remove unattached virions, followed by cell lysis and Western blotting, which served as a control to indicate the removal efficiency of attached virions. Other groups of cells were transferred to 37°C for 30 minutes to allow for internalization, either in the absence of the drug or in the presence of each candidate drug. Afterward, the cells were lysed, and the NP protein and GAPDH levels were assessed through Western blotting. **(F)** The quantitative analysis of the intensity ratio of NP to GAPDH of two Western blots using ImageJ software; The relative value of the DMSO group was set as 1. **(G)** The inhibition of influenza A virus (IAV) entry by each candidate compound is associated with the distribution of clathrin. After viral attachment (MOI = 25) at 4°C for 1 hour, cells were treated with either the candidate compound at the specified concentrations or a control. The temperature was then raised to 37°C for 1 hour to allow for internalization. Following this, the cells were fixed, permeabilized, and immunostained for nucleoprotein (NP) in green and clathrin heavy chain in red. Scale bar: 8 µm.

To clarify whether the chosen compounds impede the initial stage of viral replication, we performed an analysis to assess the completion of each step in the early phase of viral replication following drug treatment. Initially, we conducted a virus attachment assay using immunofluorescence microscopy (IFA) to detect the localization and levels of the viral nucleoprotein (NP). As shown in **Figure 5A**, treatment with each of the three compounds (isoxsuprine, ciproxifan, and rotigotine) did not affect the attachment of the influenza virus to A549 cells. In contrast, DSS, which has been reported to inhibit influenza viral attachment (Yamada et al., 2012), significantly blocked viral attachment. The quantitative analysis in **Figure 5B** further confirms that the three compounds do not affect viral attachment, while the reference molecule DSS does.

Subsequently, we employed IFA and Western blotting to evaluate the impact of the selected hit compounds on the internalization of influenza virions into A549 cells. In IFA, virions associated with or proximal to the cell membrane were excluded from the count. Because it remained ambiguous whether such virions had merely been attached to the cell membrane or internalized. Consequently, only virions that were fully encompassed within the cell membrane and not closely associated with it were quantified as internalized. The results depicted in **Figures 5C, D** demonstrate a significant reduction in internalized virions upon treatment with each of the three compounds compared to the untreated control.

To further substantiate that the administration of the compounds obstructs the internalization of the influenza virus, we assessed virion internalization through acid treatment, which eliminates attached virions, followed by western blotting to measure the quantity of viral NP, which correlates with the number of internalized virions. As indicated in **Figures 5E, F**, the data reveal that treatment with each compound significantly diminish the levels of viral NP associated with internalized virions in the treated A549 cells relative to the untreated group.

The endocytosis of the influenza virus is predominantly mediated by clathrin-mediated endocytosis (CME); however, alternative pathways such as macropinocytosis may also contribute to this process (Edinger et al., 2014; Chen and Zhuang, 2008; Luo, 2012). Research has indicated that prochlorperazine, a dopamine D2 receptor (D2R) antagonist, exhibits antiviral properties by disrupting clathrin redistribution, thereby obstructing the entry of single-stranded RNA (ssRNA) viruses (Simanjuntak et al., 2014). To investigate whether the selected drugs disrupt clathrin redistribution, we conducted immunofluorescence assays (IFA). As demonstrated in **Figure 5G**, in the absence of drug treatment, nucleoprotein (NP) and clathrin colocalization was primarily observed within the cytoplasm, indicating that the influenza A virus (IAV) enters cells via CME. In contrast, upon drug treatment, NP colocalization with clathrin was detected at the cell surface, suggesting a failure of viral entry into the cells. These findings indicate that the examined compounds inhibit IAV endocytosis by modulating CME pathways.

In conclusion, our research indicates that the compounds under investigation significantly inhibit the replication of influenza viruses by interfering with their internalization into host cells. The findings demonstrate that treatment with these selected drugs effectively obstructs clathrin-mediated endocytosis.

### 3.6 Isoxsuprine protects mice infected with the lethal influenza virus

Following our screening process, we identified several inhibitors of neurotransmitter receptors that have the potential to impede the replication of influenza viruses *in vitro*. Consequently, we conducted an *in vivo* evaluation of isoxsuprine, an adrenergic receptor agonist that exhibited lower cytotoxicity *in vitro* than the other two candidate compounds. This evaluation was carried out using a mouse model infected with a lethal strain of influenza virus to assess the protective effects of isoxsuprine against infection.

We inoculated mice with 500 TCID50 of PR8 virus, resulting in 100% mortality among infected subjects. Female BALB/c mice (6-week-old, 10 per group) received either a placebo or isoxsuprine (40, 80, or 160 mg/kg/day) 30 min before infection with mouse-adapted influenza virus A/Puerto Rico/8/1934 (H1N1). PBS containing 5% dimethyl sulfoxide (DMSO) was administered to the control group. All groups underwent treatment twice daily for three days. The mice were monitored daily for weight changes and survival over 21 days.

**Figure 6:**
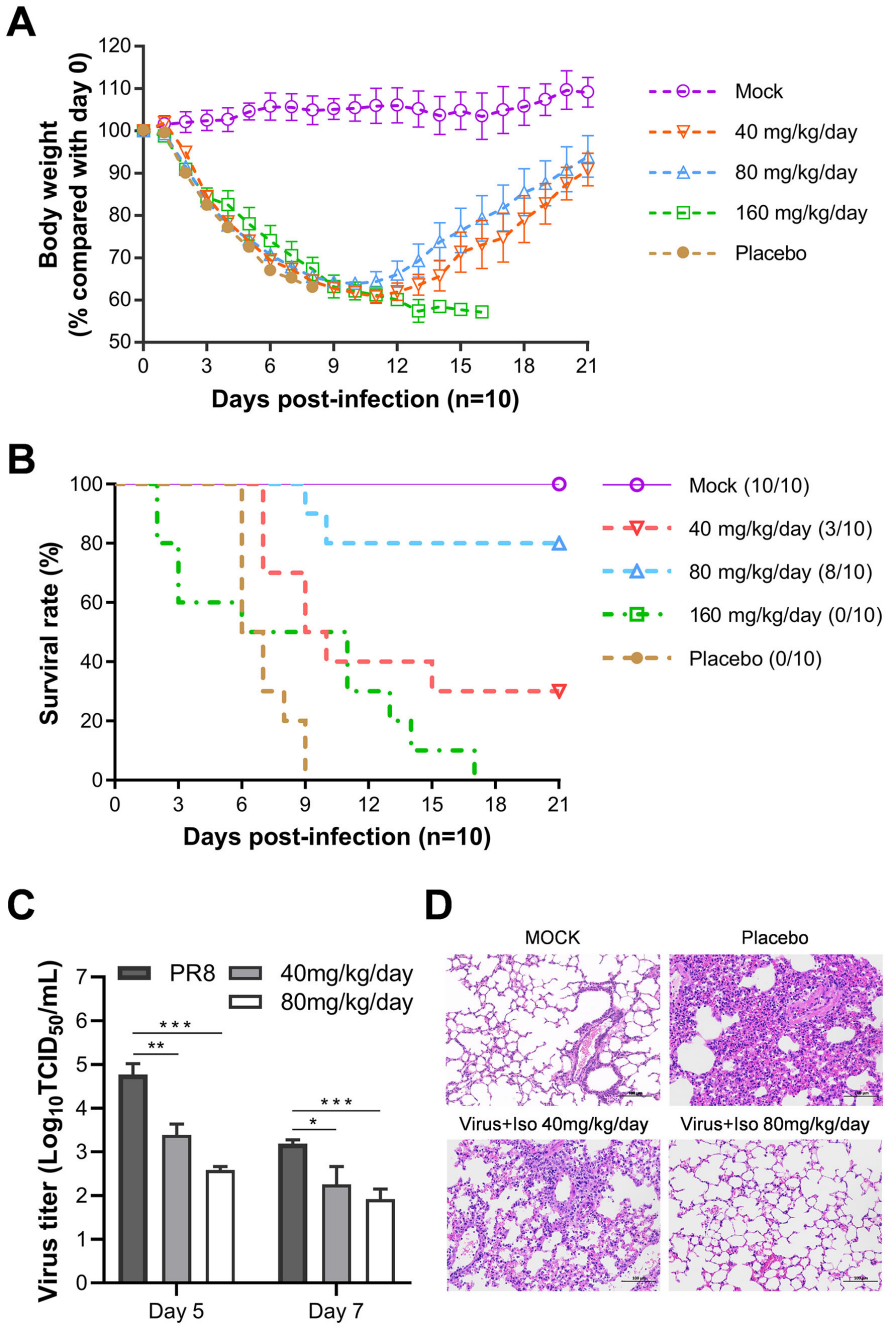
Evaluation of *in vivo* efficacy of isoxsuprine against lethal influenza infection. BALB/c mice (n=10) were infected with 500 TCID50 of PR8 influenza virus and treated with different doses of isoxsuprine or PBS containing 5% DMSO control (daily intraperitoneal injections twice a day for three consecutive days beginning 30 min pre-infection). **(A, B)** Effects of different isoxsuprine doses on body weight loss and survival rates in BALB/c mice. **(C)** Isoxsuprine administration (80 and 40 mg/kg/day) affects virus titers in the lung tissue grinding fluids of BALB/c mice (n=3). **(D)** H&E lung sections of PR8-infected BALB/c mice treated with isoxsuprine (80 and 40 mg/kg/d) or PBS controls (scale = 100 µm). Statistical significances are denoted by *P < 0.05, **P < 0.01, and ***P < 0.001.

As shown in **Figure 6A**, throughout the experiment, the body weights of all virus-infected groups continuously declined, reaching a nadir on day 11 p.i. Mice receiving the placebo succumbed within nine days. In contrast, those treated with 40 or 80 mg/kg/day isoxsuprine showed signs of weight recovery. However, the mice who were administered the highest dose of 160 mg/kg/day continued to lose weight until death, with all subjects in this group perishing by day 17 p.i.

Regarding the survival rates of the infected mice, 30% and 80% of the subjects treated with 40 and 80 mg/kg/day isoxsuprine, respectively, survived. In contrast, the group receiving 160 mg/kg/day displayed mortality starting from day 2 p.i., and all mice in this cohort died by day 17 p.i., indicating drug toxicity, which adversely affected the therapeutic benefits of isoxsuprine in addition to viral pathology (**Figure 6B**). We calculated the median survival time (T50) using the Kaplan-Meier method from GraphPad. Our results show that the T50 values for the placebo and treatment groups receiving 40 mg, 80 mg, and 160 mg/Kg/day were 6.5, 9.5, > 21, and 8.5 days, respectively. This results suggest a therapeutic effect of isoxsuprine for influenza virus infection *in vivo*.

Viral titers in the lung tissues of mice treated with 40 and 80 mg/kg/day isoxsuprine on days 5 and 7 p.i. were significantly lower than those observed in the PBS containing 5% DMSO (**Figure 6C**). In alignment with the viral replication levels, histopathological examination of lung tissues from virus-infected mice using hematoxylin and eosin (HE) staining revealed that the pulmonary parenchyma and interstitial inflammatory infiltrations in the isoxsuprine-treated groups were alleviated in a dose-dependent manner compared to those in the PBS group, with notable improvement observed in the high-dose group (**Figure 6D**).

The findings of this *in vivo* efficacy study provide compelling evidence for the protective effects of isoxsuprine treatment in mice infected with a lethal dose of influenza A virus (IAV). This protective mechanism appears to result from an antiviral effect *in vivo* and mitigation of lung damage induced by viral infection.

## 4 Discussion

Various agonists and antagonists modulate the function of neurotransmitter receptors, which play essential roles in regulating neuronal activity and behavior. The modulation of receptor function is a complex and multifaceted process. The screening library comprised antagonists (40.1%), agonists (34.1%), inhibitors (23.4%), and other reagents (2.6%) that target neurotransmitter receptors. They are pivotal in modulating neural signaling and exhibit diverse functions across different receptor types. Additionally, neurotransmitter receptors may play significant roles in non-neural cells, extending their functions beyond conventional neuronal signaling. These receptors are expressed in various non-neuronal cell types, including neural progenitors, epithelial cells, and immune cells, where they regulate a wide range of cellular processes (Belachew and Gallo, 2004; Nguyen et al., 2001; Ockenga et al., 2013). Despite the distinct characteristics of the neurotransmitter receptors, screening of this library identified 20 compounds that exhibited antiviral activity against influenza viruses. These hit compounds were distributed across the major neurotransmitter receptors targeted by the compounds in the library, achieving a hit rate of approximately 10% of the total library. Although 63.5% of the library consisted of antagonists and inhibitors of neurotransmitter receptors, only a limited number of them showed antiviral activity. This finding indicates that inhibiting the activation of neurotransmitter receptors alone is insufficient to effectively impede the replication of influenza viruses.

Three lead compounds, characterized by a higher selective index among the identified hits and functioning as antagonists or agonists of adrenergic, histamine, and dopamine receptors, were chosen for further *in vitro* efficacy studies. Our findings indicate that these three lead compounds demonstrate broad-spectrum antiviral activity against influenza A virus (IAV), influenza B virus (IBV), and oseltamivir-resistant IAV strains. Notably, these compounds exhibit marginally greater efficacy in human epithelial and monocytic cells than in canine kidney (MDCK) cells (**Table 2**). This disparity may be attributed to the fact that these compounds were initially screened in human cells, indicating that they were primarily designed for human neurotransmitter receptors. Consequently, it is plausible that they are more effective at inhibiting human neurotransmitter receptors than canine receptors are. Alternatively, variations in receptor expression levels across these cell types might contribute to the observed differences. Through a qRT-PCR analysis of the Ct (threshold cycle) values of adrenergic, histamine, and dopamine receptor genes and GAPDH gene, we found that the mRNA levels of the three receptor genes are moderate compared to that of GAPDH and slightly more abundant in U937 cells than in A549 cells (Giulietti et al, 2001) (**Supplementary Figure 1**). However, it remains unclear whether the observed antiviral effects are a result of specific on-target interactions or non-specific off-target effects. Further research is necessary to elucidate the specific drug targets and underlying mechanisms responsible for the antiviral effects.

Literature (Iwatsubo et al., 2007) shows that dopamine can induce apoptosis in young striatal neurons through D1-like receptors, but not in neonatal neurons. Meanwhile, isoproterenol, a β-adrenergic receptor agonist that increases cAMP more than dopamine, did not promote apoptosis in either group. We assessed the cytotoxicity of the three candidate drugs using an LDH assay in A549 cells treated for 48 and 72 h. Results in **Supplementary Figure 2** indicate minimal cytotoxicity at concentrations under 125 μM, with CC50 values for Iso, Cip, and Rot being 529.6, 556, and 372.1 μM, respectively. In contrast, reference drugs ethacrynic acid and etoposide have CC50 values of 240 μM and 259.7 μM, indicating more significant cytotoxicity. Higher cytotoxicity was observed at 72 h compared to 48 h. However, since antiviral experiments were conducted at concentrations below 125 μM and the mechanism study lasted only 8 h, it is unlikely that the antiviral effect resulted from cytotoxicity.

Our study examining the mechanisms of action revealed that despite targeting different neurotransmitter receptors, the selected compounds demonstrate analogous mechanisms in inhibiting viral internalization. Influenza viruses initiate infection in host cells primarily through clathrin-mediated endocytosis, the principal internalization pathway (Chen and Zhuang, 2008; Fujioka et al., 2013; Sieczkarski and Whittaker, 2002). Importantly, these viruses can penetrate cells via alternative, non-clathrin-dependent pathways (Fujioka et al., 2013; Rossman et al., 2012; Sieczkarski and Whittaker, 2002). Specifically, influenza A viruses exhibit redundant mechanisms that incorporate both clathrin-mediated and -independent endocytosis, with intracellular calcium (Ca2+) playing a crucial role in modulating both pathways (Fujioka et al., 2013). Filamentous influenza virions primarily utilize macropinocytosis for cellular entry (Rossman et al., 2012). Despite variations in cell types and multiple mechanisms of viral entry, the candidate compounds showed similar antiviral effectiveness. Our evaluation of the impact of the selected pharmacological agents on clathrin-mediated endocytosis reveals that the three candidate drugs affect the distribution of clathrin and its colocalization with the influenza viral NP. This modulation impedes the process of clathrin-mediated endocytosis.

Furthermore, certain neurotransmitter receptors display cell-type-specific signaling pathways. For instance, the serotonin receptor 5-HT1AR is involved in various pathways depending on the cell type, thereby influencing neuronal excitability, cyclic adenosine monophosphate (cAMP) levels, calcium levels, neurogenesis, and synapse formation through the MAPK and PI3K-Akt signaling pathways (Rojas and Fiedler, 2016). The internalization of influenza viruses is associated with Ca^2^+ (Fujioka et al., 2013) and PI3K-Akt signaling pathways (Saeed et al., 2008). In non-neural cell contexts, candidate compounds may impede the internalization of the influenza virus by affecting the Ca^2^+ or PI3K-Akt signaling pathways. Nevertheless, the precise mechanisms by which the selected compounds inhibit internalization of the influenza virus warrant further investigation.

Isoxsuprine hydrochloride is a specific β2-adrenergic receptor agonist that has been studied for its effects on biological systems and potential therapeutic applications. Oral cancer research has shown promise in interfering with epithelial-mesenchymal transition (EMT), which is crucial for metastasis. Treatment with isoxsuprine downregulates mesenchymal cell markers and reduces motility in oral cancer cells (Sakakitani et al., 2020). Its effects were similar to those of isoprenaline, suggesting the involvement of β-adrenergic receptor signaling. Notably, while isoxsuprine acts as an agonist, it exhibits inhibitory effects on cancer cells, indicating the complexity of adrenergic signaling. In a xenograft model, isoxsuprine effectively suppressed primary tumor growth, highlighting its potential as a cancer therapeutic agent (Sakakitani et al., 2020).

Isoxsuprine hydrochloride is a vasodilator drug that has various clinical applications. It is primarily used to reduce uterine contractions in pregnant women, potentially helping prevent preterm labor (Shahraki et al., 2020; Shiri et al., 2017). In contrast to its use in pregnancy, isoxsuprine has been shown to reduce catalase activity when binding to Bovine Liver Catalase (BLC), which could have implications for its effects on antioxidant enzymes (Shahraki et al., 2020). In conclusion, although isoxsuprine hydrochloride is primarily used in obstetrics, its applications extend to other areas of medicine. Isoxsuprine hydrochloride has been demonstrated to be safe for various clinical applications.

Our *in vivo* efficacy study of isoxsuprine, conducted using a mouse model infected with lethal influenza, revealed that isoxsuprine significantly reduced lung viral titers, alleviated pulmonary inflammation, and improved survival rates in infected subjects. Intraperitoneal administration at elevated doses (80 mg/Kg/day) raises concerns about toxicity. It is uncertain whether the antiviral effects noted can be attributed to the drug’s direct antiviral activity or are a consequence of the systemic stress induced by high doses. Furthermore, this treatment may disrupt normal physiological functions, complicating the differentiation between direct antiviral effects and potential side effects on the immune system or other vital processes. From a translational perspective, the requirement for high dosing may not be practical for human application due to safety and economic considerations. Identifying the optimal dosage and administration route is critical for ensuring the clinical viability of neurotransmitter modulators as antiviral agents. Continued research is imperative to address these limitations and validate the conclusions of this study.

Nevertheless, the findings of this study emphasize the potential of neurotransmitter receptors as novel targets in the development of antiviral agents against influenza. These results provide a foundation for optimizing and advancing identified compounds as viable therapeutic options for viral infections, including those caused by drug-resistant strains.

## Data Availability

The original contributions presented in the study are included in the article/**Supplementary Material**. Further inquiries can be directed to the corresponding authors.
