# Supplementary material for: Screening of neurotransmitter receptor modulators reveals novel inhibitors of influenza virus replication
Source: Front Cell Infect Microbiol. 2025 Apr 29;15:1562650. doi: 10.3389/fcimb.2025.1562650 (PMC12069340; doi:10.3389/fcimb.2025.1562650)
Supplement: Supplementary file 1 [file DataSheet1.pdf]

Supplemental materials

Methods for supplementary experiments

1. Real-time quantitative reverse-transcription PCR (qRT-PCR)

Total RNA extraction was performed using the Total RNA Rapid Extraction Kit (Fastagen, Shanghai, China), according to the manufacturer's protocol. Following RNA extraction, the first strand of complementary DNA (cDNA) was synthesized using SuperScript III Reverse Transcriptase (Genstar, Beijing, China). Gene-specific primers were employed in this study: GAPDH Forward (F): 5′-ATGGGGAAGGTGAAGGTCG-3′; GAPDH Reverse (R): 5′-TAAAAGCAGCCCTGGTGACC-3′; Dopamine Receptor 2 Forward (F): 5′-TCAACGGGTCAGACGGGAAGG-3′; Dopamine Receptor 2 Reverse (R): 5′-CCGAAGACGATGACAGCGATGAG-3′; β2-Adrenergic Receptor Forward (F): 5′-CGGAGCCCAGATTTCAGGATTGC-3′; β2-Adrenergic Receptor Reverse (R): 5′-CTGTGTTGCCGTTGCTGGAGTAG-3′; H3 Histamine Receptor Forward (F): 5′-GCCACTGCTATGCCGAGTTCTTC-3′; H3 Histamine Receptor Reverse (R): 5′-AGAAGGTGACGCTGAGGAAGGG-3.’ PCR amplification was conducted with 1 μL of the reverse-transcribed product using the aforementioned primers. Real-time PCR was performed using Real Star Green Power Mixtures containing SYBR Green I Dye (Genstar, Beijing, China) on a CFX96 Real-time PCR system (Bio-Rad, CA, USA). Relative mRNA expression was quantified using the $2-\Delta CT$ method, based on the cycle threshold (Ct) value.

2. Lactic Dehydrogenase Release Assay

The cells were plated in a 96-well plate and treated with or without the compound for 48 and 72 h, respectively. Following treatment, cell supernatants were collected for cytotoxicity analysis using the Lactic Dehydrogenase Release Assay Kit (GLPBIO, USA), in accordance with the manufacturer's instructions. An aliquot of 10 μL lysis solution was added to both the high control well, which contained cells and culture medium (to determine the maximum LDH release from the cells), and the high control blank well, consisting solely of culture medium (utilized for background absorbance correction). The plates were then incubated in a CO_2 incubator at 37 °C for 30 min. Subsequently, 50 μL of supernatant from each well was transferred to a new 96-well plate. An additional 50 μL of the Working Solution was added to each well of the new plate, followed by thorough mixing by shaking. The plates were then incubated at room temperature in the dark. After adding 50 μL of Stop Solution to each well, absorbance was measured immediately at 490 nm using an enzyme-linked immunosorbent assay (ELISA) reader.

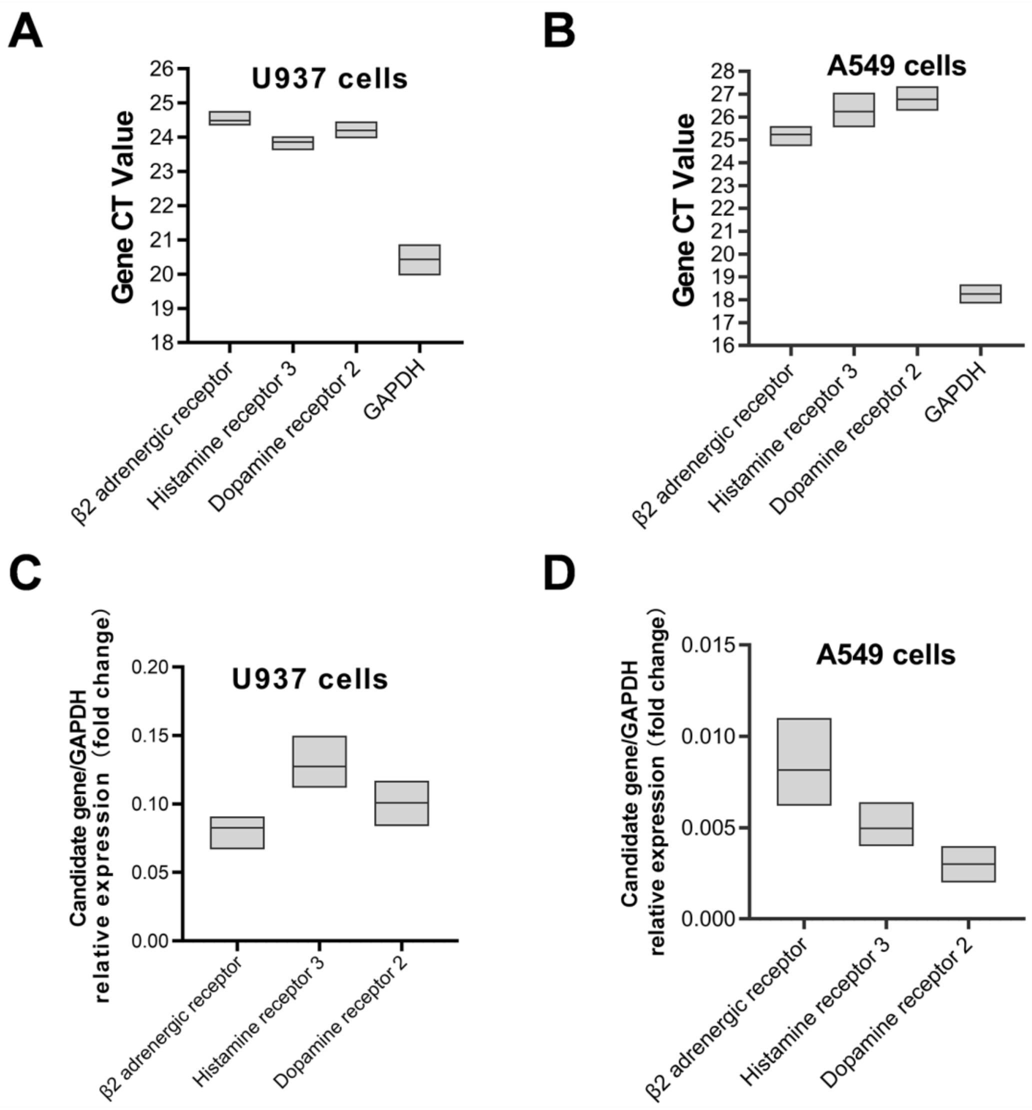

Supplemental Fig. 1. Analysis of mRNA expression levels for the three neurotransmitter receptors was conducted through quantitative reverse transcription polymerase chain reaction (qRT-PCR) in U937 and A549 cell lines. Both cell types were plated in 6-well culture plates at a density of 100,000 cells/well in DMEM supplemented with fetal bovine serum (FBS, Gibco) and 1% penicillin-streptomycin. Following a 24 h incubation period, cell lysates and supernatants were collected to facilitate total RNA extraction. The cycle threshold (CT) values obtained from qRT-PCR analysis for the β2-adrenergic receptor, H3 histamine receptor, and dopamine receptor were determined for U937 cells (A) and A549 cells (B). The relative expression of the three neurotransmitter receptors, normalized to GAPDH as a reference gene, using the 2-ΔCt method for calculating fold changes, is shown for U937 cells (C) and A549 cells (D).

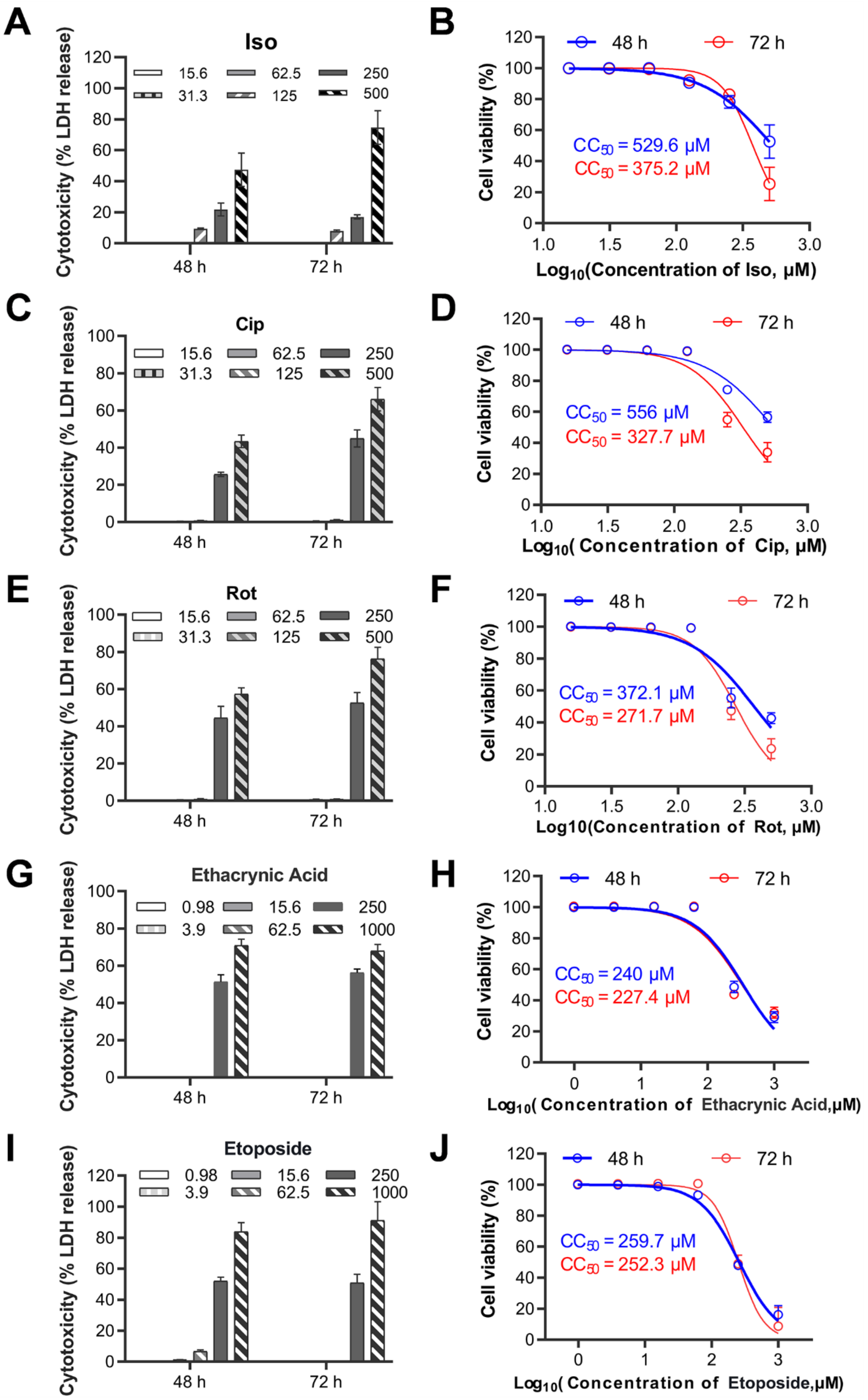

Supplemental Fig. 2. Assessment of the cytotoxicity of the three compounds in A549 Cells. A549 cells were inoculated into 96-well cell culture plates at a density of

20,000 cells/well in DMEM supplemented with fetal bovine serum (FBS, Gibco) and 1% penicillin-streptomycin. After 24 h, the culture medium was discarded and the cells were washed twice with phosphate-buffered saline (PBS). The cells were then treated with or without the test compounds for 48 or 72 h. Cell viability was evaluated using the Lactic Dehydrogenase Release Assay Kit (GLPBIO, USA). Optical density (OD) at 490 nm (OD490) was measured using a microplate reader (Thermo Fisher Scientific, MA, USA). The compounds investigated for their cytotoxic effects were isoxsuprine (A, B), ciproxifan (c, D), and rotigotine (E, F). Ethacrynic acid (G, H) and etoposide (I, J) were used as reference drugs for inducing necrosis and apoptosis, respectively. Cell viability was determined using the Lactic Dehydrogenase Release Assay Kit, with each data point representing the mean ± standard error (SD). The CC50 values were determined using GraphPad Prism software (version 8.0).
