# Supplementary material for: Screening of neurotransmitter receptor modulators reveals novel inhibitors of influenza virus replication
Source: Front Cell Infect Microbiol. 2025 Apr 29;15:1562650. doi: 10.3389/fcimb.2025.1562650 (PMC12069340; doi:10.3389/fcimb.2025.1562650)
Supplement: Supplementary file 2 [file DataSheet2.pdf]

Supplementary Table 1. Primary screening of the small-molecule compound library targeting neurotransmitter receptors

Catalog[a]	Compound Name	Target	Concentration (μM)[b]	Relative Viability[c] (%)	NA Inhibition[d] (%)
S2020	Formoterol Hemifumarate	Adrenergic Receptor	20μM	121.9	-23.1
S2090	Dexmedetomidine HCl (Precedex)	Adrenergic Receptor	20μM	119.9	-18.5
S4649	Atipamezole hydrochloride	Adrenergic Receptor	20μM	119.4	58.1
S4650	Atipamezole	Adrenergic Receptor	20μM	117.6	38.2
S2038	Phentolamine Mesylate	Adrenergic receptor	20μM	117.0	72.3
S1613	Silodosin	Adrenergic Receptor	20μM	115.2	-41.7
S2091	Betaxolol	Adrenergic Receptor	20μM	114.8	6.7
S2507	Salbutamol Sulfate	Adrenergic Receptor	20μM	114.7	6.4
S4076	Propranolol HCl	Adrenergic Receptor	20μM	114.7	65.3
S2495	Oxymetazoline hydrochloride	Adrenergic Receptor	20μM	112.6	-21.0
S1409	Alfuzosin HCl	Adrenergic Receptor	20μM	109.5	-12.3
S2092	Detomidine HCl	Adrenergic Receptor	20μM	108.7	31.2
S8114	ICI-118551	Adrenergic Receptor	20μM	106.0	60.7
S1856	Metoprolol Tartrate	Adrenergic Receptor	20μM	105.8	-24.2
S2113	Cisatracurium Besylate	Adrenergic Receptor	20μM	105.8	27.9
S4010	Acebutolol HCl	Adrenergic Receptor	20μM	105.7	-13.9
S5669	Isoxsuprine hydrochloride	Adrenergic Receptor	20μM	105.2	86.5
S2086	Ivabradine HCl	Adrenergic Receptor	20μM	104.6	-0.1
S1642	Methyldopa	Adrenergic Receptor	20μM	104.4	-13.4
S3060	Medetomidine HCl	Adrenergic Receptor	20μM	102.8	59.7
S4277	Bambuterol HCl	Adrenergic Receptor	20μM	102.5	-2.9
S2126	Naftopidil	Adrenergic Receptor	20μM	102.4	13.4
S4009	Mirabegron	Adrenergic Receptor	20μM	101.7	-9.6
S4127	Terbutaline Sulfate	Adrenergic Receptor	20μM	100.9	39.1
S4291	Labetalol HCl	Adrenergic Receptor	20μM	100.9	-17.2
S7974	L755507	Adrenergic Receptor	20μM	100.1	-1.1
S2458	Clonidine HCl	Adrenergic Receptor	20μM	99.2	-24.1
S2438	Synephrine HCl	Adrenergic Receptor	20μM	99.1	10.3
S4043	Tetrahydrozoline HCl	Adrenergic Receptor	20μM	96.0	9.5
S4123	Timolol Maleate	Adrenergic Receptor	20μM	91.2	33.2
S2362	Synephrine	Adrenergic Receptor	20μM	90.5	12.3
S4296	Salmeterol Xinafoate	Adrenergic Receptor	20μM	90.4	69.9

S1549	Nebivolol	Adrenergic Receptor	20µM	89.4	66.4
S1387	Naftopidil DiHCl	Adrenergic Receptor	20µM	88.1	6.2
S4679	Terazosin	Adrenergic Receptor	20µM	87.8	-47.2
S4124	Tolazoline HCl	Adrenergic Receptor	20µM	86.0	14.5
S1437	Tizanidine HCl	Adrenergic Receptor	20µM	78.9	-28.3
S1424	Prazosin HCl	Adrenergic Receptor	20µM	76.9	-45.6
S2499	Phenoxybenzamine HCl	Adrenergic Receptor	20µM	/[e]	95.4
S3075	Dexmedetomidine	Adrenergic Receptor	20µM	/	21.4
S3061	Epinephrine HCl	Adrenergic Receptor	20µM	/	7.1
S3185	Adrenalone HCl	Adrenergic Receptor	20µM	/	6.4
S2569	Phenylephrine HCl	Adrenergic Receptor	20µM	/	4.9
S3083	Indacaterol Maleate	Adrenergic Receptor	20µM	/	-6.0
S2566	Isoprenaline HCl	Adrenergic Receptor	20µM	/	-12.4
S2519	Naphazoline HCl	Adrenergic Receptor	20µM	/	-18.7
S2545	Scopine	Adrenergic Receptor	20µM	/	-19.4
S2516	*Xylazine HCl*	Adrenergic Receptor	20µM	/	-20.0
S2533	Ritodrine HCl	Adrenergic Receptor	20µM	/	-20.6
S2517	Maprotiline HCl	Adrenergic Receptor	20µM	/	-23.2
S2522	L-Adrenaline	Adrenergic Receptor	20µM	/	-38.4
S1283	Asenapine	Adrenergic Receptor, 5-HT Receptor	20µM	87.1	0.2
S4625	Alcaftadine	Histamine Receptor	20µM	188.5	-0.9
S2494	Olopatadine HCl	Histamine Receptor	20µM	121.5	-11.6
S1986	Meclizine 2HCl	Histamine Receptor	20µM	109.3	74.9
S2065	Lafutidine	Histamine Receptor	20µM	109.0	52.0
S1801	Ranitidine	Histamine Receptor	20µM	108.8	-11.1
S2024	Ketotifen Fumarate	Histamine Receptor	20µM	108.3	-4.7
S4008	Pemirolast potassium	Histamine Receptor	20µM	107.6	-23.7
S4293	Promethazine HCl	Histamine Receptor	20µM	105.7	64.8
S4262	Ebastine	Histamine Receptor	20µM	105.3	20.0
S3146	Tripelennamine HCl	Histamine Receptor	20µM	105.2	-0.2
S4131	Levodropropizine	Histamine Receptor	20µM	103.9	35.7
S1816	Chlorpheniramine Maleate	Histamine Receptor	20µM	102.0	1.0
S4026	*Hydroxyzine 2HCl*	Histamine Receptor	20µM	101.7	18.4
S4117	Histamine Phosphate	Histamine Receptor	20µM	98.9	-2.9
S2044	Cyproheptadine HCl	Histamine Receptor	20µM	96.7	4.6
S4696	ArbinoxaMine Maleate	Histamine Receptor	20µM	94.3	29.8
S1357	Lidocaine	Histamine Receptor	20µM	94.2	5.1
S4118	Histamine 2HCl	Histamine Receptor	20µM	93.7	2.5
S1382	Mianserin HCl	Histamine Receptor	20µM	91.9	26.3
S4139	Cyclizine 2HCl	Histamine Receptor	20µM	90.9	-4.6

S1847	*Clemastine Fumarate*	Histamine Receptor	20μM	88.5	70.1
S2813	Ciproxifan	Histamine Receptor	20μM	130.7	75.2
S2552	Azelastine HCl	Histamine Receptor	20μM	99.4	55.3
S3052	Rupatadine Fumarate	Histamine Receptor	20μM	/	22.5
S3037	Bepotastine Besilate	Histamine Receptor	20μM	/	21.1
S2905	JNJ-7777120	Histamine Receptor	20μM	/	19.7
S2585	Brompheniramine hydrogen maleate	Histamine Receptor	20μM	/	12.4
S3176	Betahistine 2HCl	Histamine Receptor	20μM	/	4.8
S3208	Fexofenadine HCl	Histamine Receptor	20μM	/	-9.9
S4053	Sertraline HCl	5-HT Receptor	20μM	124.0	68.1
S1436	Tianeptine sodium	5-HT Receptor	20μM	120.3	4.5
S2849	SB269970 HCl	5-HT Receptor	20μM	118.6	65.4
S2096	Almotriptan Malate	5-HT Receptor	20μM	114.2	21.0
S8183	Pimavanserin	5-HT Receptor	20μM	113.3	18.1
S1385	Mosapride Citrate	5-HT Receptor	20μM	111.8	32.4
S1869	Dapoxetine HCl	5-HT Receptor	20μM	110.4	63.4
S1607	Rizatriptan Benzoate	5-HT Receptor	20μM	105.8	7.7
S4109	Lorcaserin HCl	5-HT Receptor	20μM	104.4	17.8
S2025	Urapidil HCl	5-HT Receptor	20μM	103.3	5.3
S1488	*Naratriptan*	5-HT Receptor	20μM	102.2	5.8
S3050	Palonosetron HCl	5-HT Receptor	20μM	101.8	-5.5
S4693	Guanfacine Hydrochloride	5-HT Receptor	20μM	101.7	11.0
S4259	Vilazodone HCl	5-HT Receptor	20μM	98.9	-49.6
S4112	Desvenlafaxine Succinate	5-HT Receptor	20μM	98.8	11.0
S1390	Ondansetron HCl	5-HT Receptor	20μM	98.5	4.7
S8021	Vortioxetine (Lu AA21004) HBr	5-HT Receptor	20μM	95.3	23.8
S1441	Venlafaxine	5-HT Receptor	20μM	93.2	-1.3
S8010	PRX-08066 Maleic acid	5-HT Receptor	20μM	92.0	20.2
S1243	Agomelatine	5-HT Receptor	20μM	89.1	18.4
S3005	Paroxetine HCl	5-HT Receptor	20μM	85.1	51.2
S1898	Tropisetron	5-HT Receptor	20μM	80.9	55.4
S1333	Fluoxetine HCl	5-HT Receptor	20μM	80.1	10.7
S2677	BRL-15572	5-HT Receptor	20μM	/	24.4
S2691	BMY 7378	5-HT Receptor	20μM	/	23.8
S2852	BRL-54443	5-HT Receptor	20μM	/	23.6
S2860	IEM 1754 dihydrobroMide	5-HT Receptor	20μM	/	23.2
S2875	Prucalopride	5-HT Receptor	20μM	/	20.7
S2698	RS-127445	5-HT Receptor	20μM	/	20.3

S3180	Eletriptan HBr	5-HT Receptor	20μM	/	20.2
S2856	SB271046 HCl	5-HT Receptor	20μM	/	18.8
S2894	SB742457	5-HT Receptor	20μM	/	16.8
S2663	WAY-100635 Maleate	5-HT Receptor	20μM	/	9.1
S2865	VUF 10166	5-HT Receptor	20μM	/	7.6
S2582	Trazodone HCl	5-HT Receptor	20μM	/	-9.8
S2541	Clomipramine HCl	5-HT Receptor	20μM	/	-11.3
S4274	Rotigotine	Dopamine Receptor	20μM	146.8	89.1
S4289	MetoclopraMide HCl	Dopamine Receptor	20μM	126.3	-36.9
S4000	Pergolide mesylate	Dopamine Receptor	20μM	118.5	67.8
S2437	Rotundine	Dopamine Receptor	20μM	111.9	17.4
S1724	Paliperidone	Dopamine Receptor	20μM	110.3	9.3
S2451	Amantadine HCl	Dopamine Receptor	20μM	109.9	69.2
S3163	Benztropine mesylate	Dopamine Transporter	20μM	107.5	57.8
S2104	Levosulpiride	Dopamine Receptor	20μM	104.5	2.8
S1763	Quetiapine Fumarate	Dopamine Receptor	20μM	104.1	-29.1
S7993	SKF38393 HCl	Dopamine Receptor	20μM	102.3	63.2
S2011	Pramipexole dihydrochloride monohydrate	Dopamine Receptor	20μM	100.4	2.6
S2168	PD128907 HCl	Dopamine Receptor	20μM	97.5	9.1
S4639	Brexpiprazole	Dopamine Receptor	20μM	97.2	54.0
S4631	Prochlorperazine dimaleate salt	Dopamine Receptor	20μM	93.5	63.9
S2460	Pramipexole	Dopamine Receptor	20μM	91.8	-25.0
S1280	Amisulpride	Dopamine Receptor	20μM	89.7	6.2
S2529	Dopamine HCl	Dopamine Receptor	20μM	/	-7.6
S2456	Chlorpromazine HCl	Dopamine Receptor, Potassium Channel	20μM	115.0	53.3
S4031	Aclidinium Bromide	AChR	20μM	193.6	13.8
S2130	Atropine	AChR	20μM	164.2	-5.8
S4025	Homatropine Bromide	AChR	20μM	151.1	-25.8
S4292	Diphenidol HCl	AChR	20μM	124.7	32.3
S4027	Flavoxate HCl	AChR	20μM	117.7	8.2
S2087	Rivastigmine Tartrate	AChR	20μM	116.3	-0.2
S2240	Fesoterodine Fumarate	AChR	20μM	114.2	-27.7
S2490	Neostigmine Bromide	AChR	20μM	114.0	2.0
S4422	Nitenpyram	AChR	20μM	110.1	-32.4
S4619	Itopride hydrochloride	AChR	20μM	109.7	9.1
S1440	Varenicline Tartrate	AChR	20μM	108.9	6.8
S1339	Galanthamine HBr	AChR	20μM	108.7	-13.2
S2054	Orphenadrine Citrate	AChR	20μM	106.4	-8.7
S1397	Rocuronium Bromide	AChR	20μM	106.3	16.7
S4024	Homatropine	AChR	20μM	105.4	-12.0

	Methylbromide				
S4069	Hexamethonium Bromide	AChR	20μM	104.4	-10.2
S1754	Oxybutynin	AChR	20μM	104.0	-3.6
S1805	Acetylcholine Chloride	AChR	20μM	103.9	-5.4
S2455	Bethanechol chloride	AChR	20μM	102.9	2.2
S2471	Gallamine Triethiodide	AChR	20μM	102.7	-9.0
S3048	Solifenacin succinate	AChR	20μM	102.1	22.6
S4121	Succinylcholine Chloride Dihydrate	AChR	20μM	99.6	13.3
S7366	LY2119620	AChR	20μM	99.3	19.0
S2462	Donepezil HCl	AChR	20μM	99.0	4.5
S4034	Diphemanil Methylsulfate	AChR	20μM	98.2	-0.6
S1608	Pyridostigmine Bromide	AChR	20μM	88.2	-34.1
S3202	Catharanthine	AChR	20μM	/	42.3
S2659	5-hydroxymethyl Tolterodine (PNU 200577, 5-HMT, 5-HM)	AChR	20μM	/	6.5
S2508	Scopolamine HBr	AChR	20μM	/	-0.4
S3047	Otilonium Bromide	AChR	20μM	/	-2.3
S2614	Arecoline	AChR	20μM	/	-10.6
S2550	Tolterodine tartrate	AChR	20μM	/	-12.0
S2547	Tiotropium Bromide hydrate	AChR	20μM	/	-34.7
S2629	PNU-120596	AChR	20μM	/	-38.3
S3072	(R)-baclofen	GABA Receptor	20μM	112.3	46.4
S1338	Gabapentin HCl	GABA Receptor	20μM	111.9	-16.1
S2026	Ginkgolide A	GABA Receptor	20μM	108.9	23.3
S1969	Nefiracetam	GABA Receptor	20μM	104.7	6.0
S7071	(+)-Bicuculline	GABA Receptor	20μM	98.1	-11.3
S4675	Tiagabine	GABA Receptor	20μM	95.7	14.2
S2399	Dihydromyricetin	GABA Receptor	20μM	95.3	9.3
S4661	Tiagabine hydrochloride	GABA Receptor	20μM	91.7	19.9
S3018	Niflumic acid	GABA Receptor	20μM	/	23.7
S1168	Valproic acid sodium salt (Sodium valproate)	GABA Receptor, HDAC	20μM	112.7	-18.5
S4688	Perampanel	GluR	20μM	98.8	-30.9

S7072	NMDA (N-Methyl-D-aspartic acid)	GluR	20μM	112.9	-15.5
S2690	ADX-47273	GluR	20μM	/	34.4
S2809	MPEP	GluR	20μM	/	32.6
S2861	CTEP (RO4956371)	GluR	20μM	/	31.0
S2795	VU 0357121	GluR	20μM	/	29.7
S2862	VU 0364770	GluR	20μM	/	28.5
S2876	(-)-MK 801 Maleate	GluR	20μM	/	26.1
S2892	VU 0361737	GluR	20μM	/	23.5
S2857	MK-801 (Dizocilpine)	GluR	20μM	/	20.9
S2103	Naltrexone HCl	Opioid Receptor	20μM	107.1	10.1
S2085	Trimebutine	Opioid Receptor	20μM	107.6	43.7
S2322	(+)-Matrine	Opioid Receptor	20μM	109.9	9.9
S4501	Naloxone HCl Dihydrate	Opioid Receptor	20μM	113.1	27.3
S2480	Loperamide HCl	Opioid Receptor	20μM	115.9	23.2
S2722	JTC-801	Opioid Receptor	20μM	/	41.4
S3066	Naloxone HCl	Opioid Receptor	20μM	/	25.4
S2503	Racecadotril	Opioid Receptor	20μM	/	-29.5

a. Catalog numbers of selected compounds from Selleck Chemicals (Shanghai, China)

b. Relative cell viability (%) was used to preliminarily assess the toxicity of the compounds, and it was calculated as: $OD_{(490-630)}$ of compound-treated group / $OD_{(490-630)}$ of virus-infected group × 100

c. 197 compounds were added to the assay plate via an acoustic liquid handling system at a final concentration of 20 μM. See the Materials and Methods section for details.

d. The inhibition rate of influenza virus neuraminidase activity was used to preliminarily assess the antiviral activity of the compounds. It was calculated using the following formula: 100 - [(fluorescence value of the drug-treated group - fluorescence value of the blank) / (fluorescence value of the virus-infected group - fluorescence value of the blank)] × 100

e. The symbol "/" indicates missing data due to experimental limitations, undetectable results, or color interference in the cell viability assay.
